# A dorsalis pedis venous flap containing a U-shaped venous arch for the reconstruction of fingertip defects

**DOI:** 10.1177/17531934221098064

**Published:** 2022-05-02

**Authors:** Li Ling, Xueyuan Li

**Affiliations:** 1 Department of Hand Surgery, Keqiao Chinese Medicine Hospital, Shaoxing, China; 2 Department of Hand Surgery, Ningbo No.6 Hospital, Ningbo, China

**Keywords:** Fingertip defects, venous flap, arterialized, dorsalis pedis

## Abstract

In small-area soft tissue defects of the fingers, venous flaps have the advantages of easy design and harvesting, and also a thin and flexible texture. Although the artery–vein–vein (A–V–V) pattern is the safest pattern, the tiny veins in the finger pulp make anastomosis difficult. We have designed a modified blood flow pattern flap using the dorsum of the foot as the donor site and anastomosing the U-shaped blood vessels in the venous flap with the two digital arteries in an artery–vein–artery (A–V–A) pattern to reconstruct the digital arterial arch without the need for venous anastomosis. Our study included 22 patients with 23 fingertip defects. In the 23 fingers, 18 flaps survived completely and four had only partial marginal necrosis. The donor site healed well without significant scar hyperplasia.

**Level of evidence:** IV

## Introduction

Fingertip skin and soft tissue defects are some of the most common hand injuries. A wide variety of flaps have been reported for their repair, according to the extent of the defect; examples include the pedicled island flap, adjacent finger flap, advancement flap and free flap. Owing to the constraints of the different techniques, traditional operations may result in residual scars, various complications, reoperation and other problems (Casoli et al., 2004; Georgescu et al., 2007; Kawakatsu and Ishikawa, 2010; Pham and Netscher, 2015). In particular, the free perforator flap or toe flap is technically demanding. Among the many flaps, arterialized venous flaps have gained attention from surgeons owing to their hidden donor sites, convenient harvesting and satisfactory survival rate (Kawakatsu et al., 2013; Yang et al., 2016). Venous flaps were first described by Nakayama et al. (1981), who demonstrated through animal experiments that the distal end of the venous flap can be nourished when it is perfused with arterial blood. The arterialized venous flap is one of the various flaps with a non-physiological blood supply that have emerged in the field of microsurgery. With the wide application of venous flaps in clinical practice, the survival rate has reached 89–100% (Goldschlager et al., 2012; Lin et al., 2010). These venous flaps can survive excellently in different arterial perfusion patterns (artery–vein–artery (A–V–A) or artery–vein–vein (A–V–V)) and can be used to successfully repair various types of finger defects (Goldschlager et al., 2012; Rozen and Leong, 2012).

Since fingertip defects cannot be repaired by the classic A–V–V pattern owing to the lack of proper finger pulp veins, we have designed a dorsalis pedis venous flap containing a U-shaped venous arch to provide a blood supply in an A–V–A pattern using the two digital arteries, which is equivalent to reconstructing the distal digital arterial arch. Because the vein in the flap is anastomosed with both the digital arteries, it is different from the traditional unilateral A–V–A pattern, which was a flow-through pattern, and there is no need to anastomose the return vein. In this article, we focus on the surgical technique of the bilateral A–V–A pattern and discuss its clinical feasibility, advantages and disadvantages.

## Methods

### Patients

**Figure 1.**
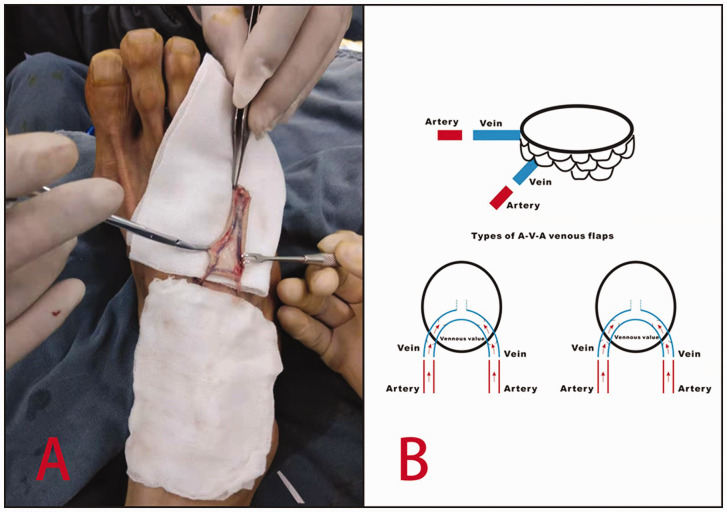
Surgery planning. (a) A clearly visible venous network in the flap from the dorsum of the foot and (b) The blood supply and different directions of the venous valves.

This study was approved by the ethics committee of Keqiao Traditional Chinese Medicine Hospital. From 2018 to 2020, 22 cases with 23 soft tissue defects of the finger pulp were repaired with venous flaps. There were 15 men and seven women, with a mean age of 41 years. Eight patients had a history of smoking 10–20 cigarettes per day. There were three thumb injuries, eight index finger injuries, seven middle finger injuries, four ring finger injuries and one little finger injury. The mean defect area was 1.8 cm × 2.2 cm. In all cases, the dorsum of the foot was selected as the donor site. An appropriate venous network was found in the dorsum of the foot and a flap was designed (Figure 1). The vein in the flap was directly anastomosed with the proximal ends of both digital arteries with an A–V–A blood supply pattern. The first 16 cases the veins for anastomosis were in the proximal edge of the flap so the flap did not need to be turned 180°; in the next five cases the vessels were in the distal edge so the flap needed to be turned 180°; and one case was a two-finger defect, with a reverse blood supply in the venous flap for one finger (the flap did not need to be turned 180°) and a forward blood supply in the venous flap for the other (the flap needed to be turned 180°). In all cases, an end-to-end dorsalis pedis nerve was attached to a digital nerve to facilitate flap sensation in the later stage.

### Surgical technique

With the patient in the supine position, brachial plexus anaesthesia was followed by abduction of the upper limb and placement of a pneumatic tourniquet. A pneumatic tourniquet was placed on the ipsilateral lower limb, and the dorsal foot incision was made under local anaesthesia. The flap was selected in the region between the metatarsal head and the tarsometatarsal joint, where the superficial veins have a good match with the vessel diameter in the recipient site. After debridement of the recipient site, the proximal ends of the digital arteries and the one digital nerve were identified under the microscope. The flap was designed according to the defect area. The venous network needed to be evenly distributed in the flap (Figure 1), and the un-needed branch of the vein was ligated, leaving only two divided ends. The flap was elevated from the superficial fascia to ensure that the flap was thin, while an appropriate length of the dorsal cutaneous nerve of the foot was harvested. The wound at the donor site was closed, compressed and bandaged in one stage. The divided ends of both digital arteries in the affected area were trimmed until they were healthy. They were flushed with heparin solution. The venous flap was placed in the defect site of the recipient area according to the direction of the U-shaped vessel and the wound margin was sutured with 5-0 absorbable sutures. Under the microscope, 10-0 nylon sutures were used to directly anastomose each digital artery with the U-shaped vein in the flap to form an A–V–A pathway. The dorsalis pedis nerve was joined to one digital nerve. The upper-limb tourniquet was released, and the flap was observed until its colour turned pink. The operation took around 70–90 minutes.

### Postoperative care

The patient stayed in bed for 5–7 days after the operation. The flap oedema reached a peak at 3–4 days; when tension blisters occupied half of the flap area, the congestion in the epidermis of the flap needed to be extruded by puncture with a needle. Anticoagulation (low molecular heparin) and anti-infection treatments were given. Patients were kept warm by putting a heat lamp 30–40 cm above them. All patients started physical therapy after 2 weeks. All patients were advised to avoid smoking for 6 months after discharge.

### Outcome assessment

The patients were followed-up for a mean of 12 months (range 9–15) after operation to observe six indicators: flap survival; late-stage pigmentation; secondary contracture; complications at the donor site; function and aesthetic appearance of the affected finger; and two-point discrimination (2-PD).

## Results

**Figure 2.**
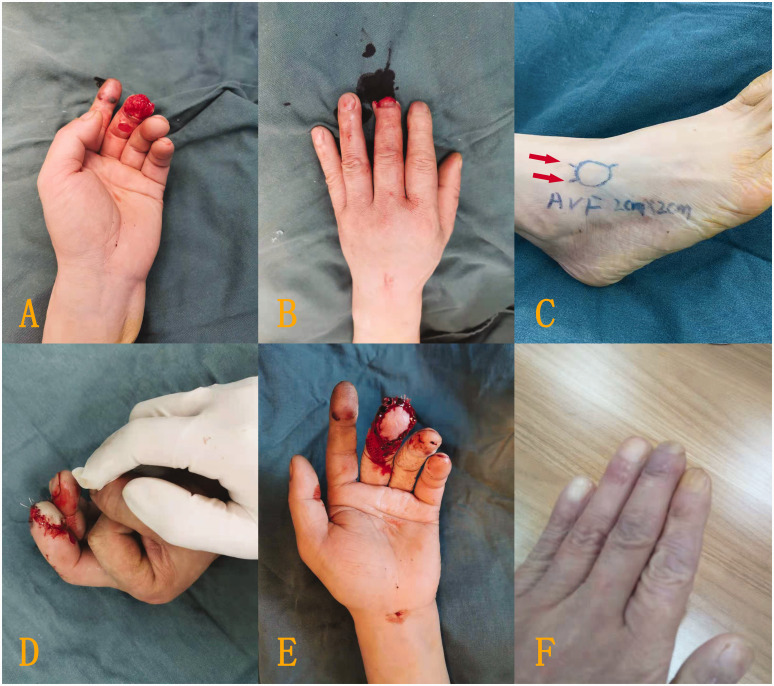
Typical case with left middle finger tissue defect. (a, b) The defect. (c) A venous flap was designed on the dorsum of the foot according to the defect area, and the proximal ends of the vein were anastomosed to the divided ends of the bilateral digital arteries to form a reverse blood supply in the venous flap. The red arrow represents the direction of blood supply. (d) After the flap covered the wound surface and the blood vessels were anastomosed, the colour of the flap was satisfactory. (e) The flap successfully survived after 1 week, with good colour and a dry wound surface and (f) The digit had a satisfactory appearance and normal colour at longer follow-up.

**Figure 3.**
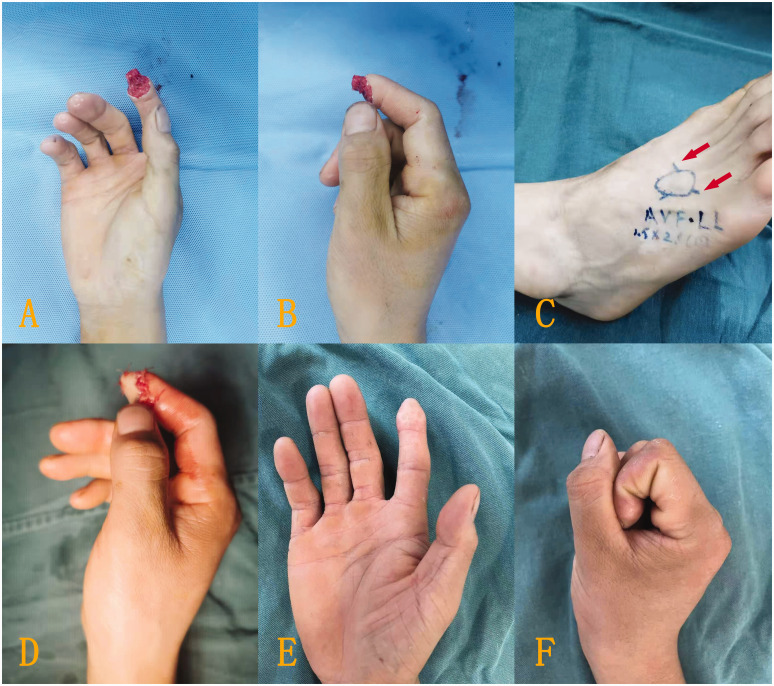
Typical case with right index fingertip soft tissue defects. (a, b) The defect. (c) A venous flap was designed on the dorsum of the foot according to the defect area, and the distal two ends of the vein were anastomosed to the divided ends of the bilateral digital arteries to form a forward blood supply in the venous flap. The red arrow represents the direction of blood supply. (d) The flap covered the wound surface after rotation through 180° and (e, f) The flap had a satisfactory appearance and normal colour after survival and fist clenching and finger pinching were normal.

**Figure 4.**
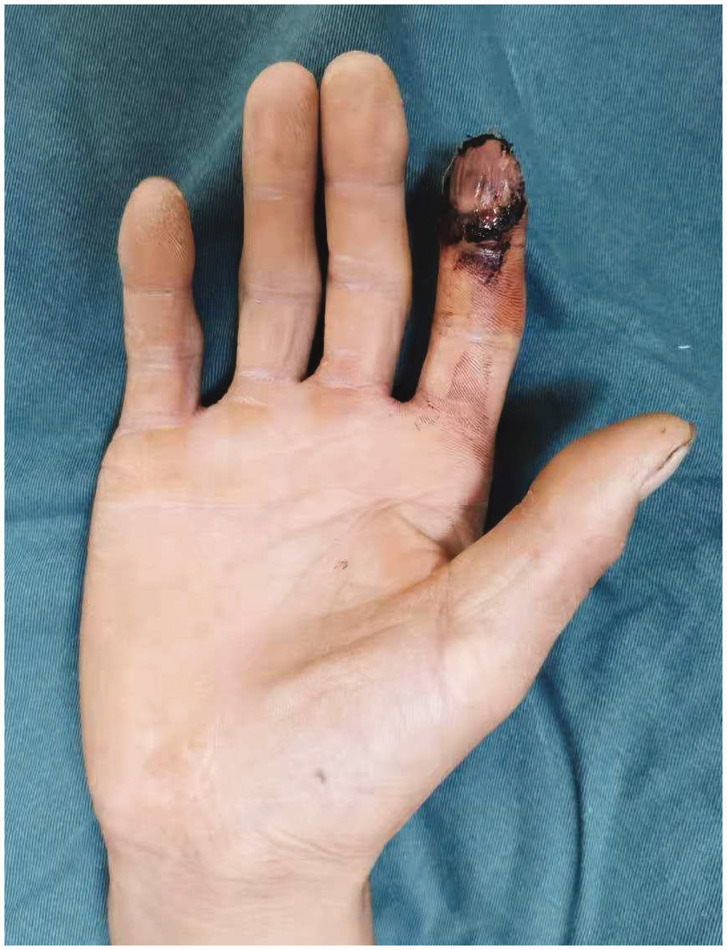
Partial necrosis with an area of about 5% in the flap edge. There was significant exudate at the wound edge. The necrotic edge healed by itself.

Of the 23 digits, the flaps of 18 digits survived successfully with either reverse (Figure 2) or forward blood supply (Figure 3). Four patients developed partial necrosis at the edge of the flap after 6 days (Figure 4), with necrotic areas between 4–6%. The necrotic part adhered to the scab at the wound margin. After removing the surrounding scab and part of the necrotic tissue, the tissue healed after application of a local wet compress with heparinized saline gauze, though a mild secondary contracture occurred at the skin margin later. Five flaps developed small tension blisters 3–4 days after the surgery, with single blisters and multiple sporadic blisters, three cases of which were associated with mild pigmentation during long-term follow-up. Venous flaps with a forward blood supply tended to be swollen and congested and were prone to the development of small blisters after operation. One week later, the flaps had survived, but the entire wound surface had a small amount of exudate. Venous flaps with a reverse blood supply had a slightly slower postoperative congestion process, and the flaps and the wound margins were drier.

All fingers regained full range of motions. The mean 2-PD discrimination in the flaps was 5.6 mm (range 4.0–7.7) after a mean follow-up of 12 months. The donor site of the dorsalis pedis healed well without significant scar hyperplasia and all patients were satisfied with the functional and aesthetic results.

## Discussion

In this case series, we repaired digital pulp defects with a venous flap with U-shaped vein arch from the dorsum of the foot and achieved satisfactory results. This specially designed U-shaped vein simulates the arterial arch of the finger pulp and nourishes the micro-vessels of the venous network through pressure difference to achieve blood supply for the flap (Lam et al., 2013). All flaps survived without venous drainage. The survival rate of the venous flaps in this group was similar to or better than the results of venous arterialized flaps reported by Garlick et al. (2015) and De Lorenzi et al. (2002).

Chen et al. (1991) first proposed the classification of venous flaps. According to this classification arterialized venous flaps (Type III) were those with the highest survival rate. They also studied the direction of the venous valves and arterial flow and found no effect on flap survival. Woo and Seul (2001) also reported similar results. In our group, in 17 digits, the arterial blood flowed through reversed venous valves, and the postoperative capillary refill was slow, the skin flap was slightly pale and the wound surface was clean. In the other six digits, arterial blood flowed through forward venous valves, the skin flap was ruddy in colour, with speedy capillary refill and increased tension in the tissue. Small blisters were found on the third to fourth days after surgery and a small amount of wound exudation occurred within 1 week after surgery. Finally, all flaps survived with no differences between the directions of the valves.

The A–V–A mode of venous flaps is a subtype of the Type III of Chen et al. (1991). Goldschlager et al. (2012) made a modification of the classification of Chen et al. in which the A–V–A mode was classified as Type IV. The arterialized venous flow-through flap is now widely used for replantation of amputated digits (Liu et al., 2014) with arterial and skin defects or for release of severe scar contracture after digital burn injuries (Iwuagwu et al., 2013). The vein in the flap is anastomosed with one digital artery proximally and distally to restore the continuity of the digital artery. The flap does not require anastomosis with a draining vein, but the distal arterial blood can return through other subcutaneous veins. Koch et al. (2004) and Liu et al. (2014) believed that the A–V–A mode, with a forward-flowing arterialized vein, had the risk of insufficient perfusion at the edge of the flap, causing blisters or partial necrosis.

In our series, the U-shaped vein arch was anastomosed with the two digital arteries, which is different from the Type III A–V–A mode of Chen et al. In terms of survival mechanism, we hypothesize that the arterial pressure that drives the blood flow with a to-and-fro mode in the capillaries contributed to flap survival. Whenever the venous pressure exceeded the intraluminal pressure, the venous blood was drained by the arteries (Lin et al., 2004). Imanishi et al. (1996) indicated that digital arteries can push blood into the venous vascular network through pressure difference. Chen et al. (1991) believed that small-sized venous flaps had a better survival rate. The venous flap harvested from the dorsum of the foot in our group was small and super thin; moreover, the exudation at the incision edge could help relieve venous congestion, which was also one of the reasons for the high survival rate.

Recently, there have been several reports about sensory restoration in venous flaps by attaching a subcutaneous nerve. The 2-PD achieved was between 6–14 mm (Lee et al., 2019; Zhu et al., 2014). Kushima et al. (2002) pointed out that no sensory recovery has been recorded in those venous flap harvested from the forearm or dorsum of the foot. In this study, we joined a subcutaneous nerve in the flap from the dorsum of the foot to a digital nerve and the 2-PD was better than the results noted above. However, this might not necessarily be the result of nerve repair as residual sensory receptors in the fingertip cannot be excluded (Yan et al., 2012).

The venous flap with a U-shaped vein network from the dorsum of the foot has several advantages in repairing fingertip defects. The dorsum of foot is normally covered from view. It contains an abundant venous plexus and it is easy to find U- or V-shaped venous arches. The calibre of the dorsal foot veins matches that of digital artery, thus facilitating the vascular anastomosis. The flap is easy to dissect, and the harvesting time is only around 15 minutes. Compared with the free perforator flap or free toe flap, venous flaps have less operating time (Choi et al., 2020). Type III venous flaps have a relatively high survival rate and few complications, especially with regard to postoperative atrophy and pigment depositions (Kushima et al., 2002), which were not obvious in our cases. The dorsal cutaneous nerve can be used to restore sensation in the fingertip.

Although this flap has many advantages, it also has some disadvantages. The dorsum of the foot does not possess glabrous skin, unlike the free toe flap or hypothenar flap. Venous flaps have more transient problems, such as increased tension in the flap, oedema and blisters after surgery, which require careful observation and timely management. Moreover, the applicability of this special type of venous flap is uncommon, and the survival mechanism of the flap without venous drainage is still unclear.
